# Novel Bi-Allelic Variants of *FANCM* Cause Sertoli Cell-Only Syndrome and Non-Obstructive Azoospermia

**DOI:** 10.3389/fgene.2021.799886

**Published:** 2021-12-15

**Authors:** Yuxiang Zhang, Peng Li, Nachuan Liu, Tao Jing, Zhiyong Ji, Chao Yang, Liangyu Zhao, Ruhui Tian, Huixing Chen, Yuhua Huang, Erlei Zhi, Ningjing Ou, Haowei Bai, Yuchuan Zhou, Zheng Li, Chencheng Yao

**Affiliations:** ^1^ Depart. of Andrology, Center for Men’s Health, Urologic Medical Center, Shanghai General Hospital, Shanghai Jiao Tong University School of Medicine, Shanghai, China; ^2^ Shanghai Key Lab of Reproductive Medicine, Shanghai Jiao Tong University School of Medicine, Shanghai, China; ^3^ State Key Lab of Reproductive Medicine, Nanjing Medical University, Nanjing, China; ^4^ The International Peace Maternity and Child Health Hospital, Shanghai Jiao Tong University School of Medicine, Shanghai, China

**Keywords:** *FANCM*, gene mutation, male infertility, Sertoli cell-only syndrome, non-obstructive azoospermia

## Abstract

Non-obstructive azoospermia (NOA) is the most severe disease in male infertility, but the genetic causes for the majority of NOA remain unknown. FANCM is a member of Fanconi Anemia (FA) core complex, whose defects are associated with cell hypersensitivity to DNA interstrand crosslink (ICL)-inducing agents. It was reported that variants in *FANCM* (MIM: 609644) might cause azoospermia or oligospermia. However, there is still a lack of evidence to explain the association between different *FANCM* variants and male infertility phenotypes. Herein, we identified compound heterozygous variants in *FANCM* in two NOA-affected brothers (c. 1778delG:p. R593Qfs*76 and c. 1663G > T:p. V555F), and a homozygous variant in *FANCM* (c. 1972C > T:p. R658X) in a sporadic case with NOA, respectively. H&E staining and immunohistochemistry showed Sertoli cell-only Syndrome (SCOS) in the three patients with NOA. Collectively, our study expands the knowledge of variants in *FANCM*, and provides a new insight to understand the genetic etiology of NOA.

## Introduction

Infertility is a common reproductive disorder affecting about 8–12% of couples worldwide. Male-infertility-associated factors are found in approximately half of these cases (Agarwal et al., 2021). Azoospermia, which is defined as the complete absence of spermatozoa in the ejaculate, and accounts for 10–15% of male infertility cases. In terms of azoospermia, 70% of cases represent non-obstructive azoospermia (NOA) with the absence or reduction of germ cells owing to testicular atrophy (Stephen and Chandra, 2006). Sertoli cell-only Syndrome (SCOS) is the most severe type of NOA with impairment of spermatogenesis, which is characterized as complete loss of male germ cells in the seminiferous tubules with only Sertoli cells retained (Ezeh et al., 1998).

NOA is heterogeneous in etiology, including Y chromosome microdeletions, chromosomal abnormalities, hypogonadism, testicular tumor, cryptorchidism, varicocele, improper drug administration, and single gene mutations (Minhas et al., 2021). Recently, several causative genes have been identified by whole-exome sequencing (WES) in NOA pedigrees, such as DNA Meiotic Recombinase 1 (DMC1 MIM: 602721), Stromal Antigen 3 (STAG3 MIM:608489), Testis Expressed 11 (TEX11 MIM: 300311), Shortage In Chiasmata 1 (SHOC1, MIM: 618038), Synaptonemal Complex Central Element Protein 1 (SYCE1 MIM: 611486), Meiosis Specific With OB-Fold (MEIOB MIM: 617670), Coiled-Coil Domain Containing 155 (CCDC155 MIM: 618125), Testis Expressed 14 (TEX14 MIM: 605792), Testis Expressed 15 (TEX15 MIM: 605795), and X-Ray Repair Cross Complementing 2 (XRCC2 MIM: 600375) (Maor-Sagie et al., 2015; Okutman et al., 2015; Yang et al., 2015; Gershoni et al., 2017; Fakhro et al., 2018; He et al., 2018; Yang et al., 2018; Riera-Escamilla et al., 2019; Minhas et al., 2021; Yao et al., 2021). Recently, it was demonstrated that compound heterozygous *FANCM* variants (c. 1491dupA: p. Gln498Thrfs*7 and c. 4387–10A > G: p. Arg1436_ Ser1437insLeuLeu*) were identified in two NOA-affected brothers in an Estonian family. Histopathological analysis showed SCOS in the proband. Another two homozygous variants (c. 5101C > T: p. Gln1701* and c. 5791C > T: p. Arg 1931*) were identified in two sporadic NOA-affected cases (Kasak et al., 2018). Furthermore, bi-allelic *FANCM* pathogenic variants (PVs) were also reported in oligoasthenospermic or azoospermic patients. It was reported that a homozygous *FANCM* frameshift variant (c. 1946_1958del: p. P648Lfs*16) was identified in three brothers with idiopathic infertility in a family. Two affected cases were diagnosed with oligoasthenospermia, whereas another was diagnosed with azoospermia (Yin et al., 2019). However, there is still no evidence to explain the association between different types of *FANCM* variants and male infertility phenotypes.

The present study reports on three patients with SCOS and NOA due to novel bi-allelic variants in *FANCM*. Compound heterozygous *FANCM* variants (c. 1778delG: p. R593Qfs*76 and c. 1663G > T: p. V555F) were identified in two NOA-affected brothers. Another homozygous *FANCM* variant (c. 1972C > T: p. R658X) in a sporadic NOA-affected patient. Hematoxylin and eosin (H&E) and immunofluorescence (IF) staining showed SCOS in all three NOA-affected patients. Together, our study ascertained *FANCM* as the candidate gene for SCOS, and provided novel foci for NOA genetic counseling.

## Materials and Methods

### Subjects

All three patients with male infertility were diagnosed with idiopathic NOA at the Department of Andrology, Center for Men’s Health, Urologic Medical Center, Shanghai General Hospital, Shanghai Jiao Tong University School of Medicine. Family histories were collected. Furthermore, 479 patients with sporadic NOA-affected patients were enrolled in this study. Reproductive congenital diseases such as Klinefelter syndrome and genomic AZF deletions, or other azoospermia-associated factors including varicocele, radiation, chemotherapy, orchitis, cryptorchidism, and testicular cancer were excluded for these patients with NOA.

This study was approved by the Institutional Ethical Review Committee of Shanghai General Hospital, Shanghai Jiao Tong University (Permit Number: 2020SQ199). Written informed consent was obtained from the individual(s) and/or minor(s) legal guardian/next of kin for the publication of any potentially identifiable images or data included in this article.

### WES and Variant Filtration

Genomic DNA was extracted from blood samples using the DNeasy Blood and Tissue Kit (Qiagen). The quality and size of libraries were measured by 2,100 Bioanalyzer High Sensitivity DNA Assay (Agilent Technologies).

For next-generation sequencing, the qualified libraries were applied to 2 × 150-bp paired-end sequencing on the Illumina NovaSeq platform (Illumina, San Diego, and United States). Raw data files were obtained from Novaseq 6,000 and then were demultiplexed and converted to fastq format using bcl2fastq software for downstream analysis. Adapters and reads with low quality were trimmed using fastp software. The BAM files were obtained by aligning the sequence reads to the reference (hg19/GRCH37, fasta format) with the use of the SpeedSeq. Additionally, duplicate reads were flagged in the BAM files to prevent downstream variant call errors, sample contamination, and swaps using VerifyBamID. Then the UnifiedGenotyper tool of GAT was used to call SNVs. The variants were annotated using Annovar software. During the annotation, several public databases such as Clinvar, gnomAD, and dbNSFP, *etc.* were used. Variants with allele frequencies higher than 1% in any public databases (ExAC Browser and gnomAD) were excluded. Because autosomal recessive or X-linked inheritance was assumed for spermatogenic defect, genes with two alleles of potentially deleterious missense mutations (SIFT, PolyPhen-2, and MutationTaster), or loss-of-function (LoF) variants were kept for further analysis. Moreover, we compared candidate genes with human germline-enriched genes in the database and known pathogenic genes for azoospermia in mice. The aforementioned sequencing and bioinformatic analyses were conducted together with the Nuprobe company (Shanghai, China). The datasets used and analyzed during the current study are available from the corresponding author on reasonable request.

### Sanger Sequencing

Validation of *FANCM* variants in the patients with NOA was performed with Sanger sequencing. Genomic DNA was extracted from peripheral blood using the TIANamp Genomic DNA Blood Kit (TIANGEN, China, and Beijing) according to the manufacturer’s instructions. The exon regions of *FANCM* were amplified with PCR primers (Supplementary Table S1). The PCR products were bidirectionally sequenced through a 3730xl DNA Analyzer (Applied Biosystems, California, and United States).

### H&E Staining

The testicular biopsies were obtained from the NOA-affected cases and the obstructive azoospermia (OA)-affected patient as the control. The testicular tissues were fixed in 4% paraformaldehyde solution overnight, embedded in paraffin, and sectioned at 5 μm thickness. Paraffin sections were dewaxed with xylene for 30 min, and then rehydrated sequentially in 95, 90, 85, and 70% ethanol each for 10 min, submerged in Phosphate-Buffered Saline (PBS) solution for 10 min, and stained with hematoxylin and eosin according to standard protocols (catalogue number: ab245880, Abcam, Cambridge, and United Kingdom). All the staining sections were captured by phase-contrast microscope to observe the structural changes of testicular tissues (Leica).

### Immunofluorescence (IF) Staining

The testis tissues were dewaxed in xylene and rehydrated in descending alcohol. Antigen retrieval was performed by incubation with 10 mM sodium citrate pH 6.0, 0.05% Tween-20 at 90–98°C for 30 min. The slides were cooled to room temperature and washed with PBS-T (PBS + 0.5% Tween-20). Non-specific antibody binding was blocked in 5% donkey serum for 1 h at room temperature and then incubated overnight with anti-DEAD-Box Helicase 4 (DDX4; dilution: 1:500; catalogue number: ab13840, Abcam), anti-Ubiquitin C-Terminal Hydrolase L1 (UCHL1; dilution: 1:500; catalogue number: ab8189, Abcam), anti-Androgen Receptor (AR; dilution: 1:100; catalogue number: sc-7305, Santa Cruz), and anti-SRY-Box Transcription Factor 9 (SOX9; dilution: 1:400; catalogue number: AB5535, Millipore) at 4°C in a humidified chamber. After three washes in PBS-T, secondary antibodies were applied for 1 h at room temperature. Subsequently, the slides were washed three times in PBS-T and mounted with antifade medium with 4,6-diamidino-2-phenylindole (DAPI; Vector). The images were captured by a fluorescence microscope (Leica).

## Results

### Clinical Characteristics

**FIGURE 1:**
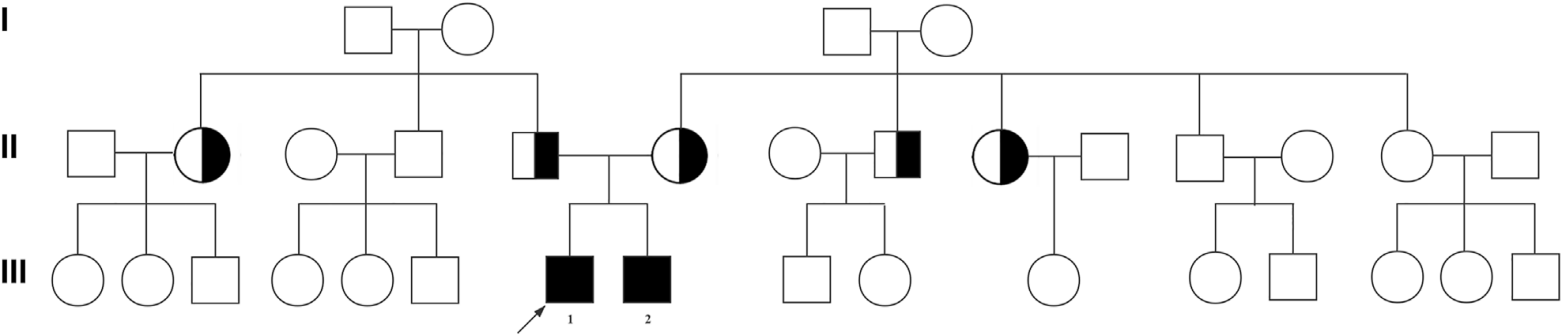
Pedigree of the affected family with *FANCM* variants. Family pedigree with black fill denoting NOA-affected patient P6649 (Ⅲ-1) and his elder brother (Ⅲ-2). The arrowheads indicate the probands.

The two brothers and the sporadic patient were diagnosed with idiopathic NOA at our center. There was no family history of consanguinity or fertility problems and no chronic diseases in all three NOA-affected cases (Figure 1). None of the patients had a history of cryptorchidism, hypogonadism, cancer, and drinking, or smoking. Physical examination revealed normal development of penis, epididymis, prostate, scrotum, and vas deferens. There was no varicocele in the three patients.

The two brothers (P6612, Ⅲ-1 and P6612-B, Ⅲ-2) had a history of male infertility for 4 and 5 years respectively. Physical examination showed the small size of the testis (10 ml in the proband, and 8 ml in the elder brother, both sides). Routine semen analyses (three times) revealed normal volume but no sperm in the ejaculate, indicating complete azoospermia. Laboratory examination revealed elevated FSH levels (14.29 IU/L and 12.85 IU/L versus 1.5–12.5 IU/L in normal). Similarly, for another sporadic NOA-affected case (P6612) with a history of male infertility for 2 years (23 years old), the results of physical examination and semen analyses (three times) suggested normal testis volume (12 ml, both sides), however, complete azoospermia. Laboratory examination also revealed elevated FSH levels (24.95 IU/L versus 1.5–12.5 IU/L in normal). All patients have 46,XY karyotypes without microdeletions in the Y chromosome. The clinical characteristics mentioned above are summarized in Table 1.

**TABLE 1:** Clinical and semen characteristics in patients with bi-allelic *FANCM* variants.

Characteristics	Subject			
	P6649	P6649-B	P6612	Reference
Age (years)	31	34	23	—
Testicular Volume (Left) (ml)	10	8	12	12–15
Testicular Volume (Right) (ml)	10	8	12	12–15
FSH (IU/L)	14.29	12.85	24.95	1.27–19.26
LH (IU/L)	8.12	7.99	12.77	1.24–8.62
T (μg/L)	4	4.73	1.75	1.75–7.81
Karyotype	46,XY	46,XY	46,XY	46,XY
Y Chromosome Microdeletions	N	N	N	N
Semen parameters				
Semen volume (ml)	1.6	2.1	2.6	≥1.5
Sperm concentration (10^6^/ml)	0	0	0	≥15
PR (%)	0	0	0	≥32
NP (%)	0	0	0	—
IM (%)	0	0	0	—
Centrifuged spermatozoa number (/ejaculate)	0	0	0	—

FSH, follicle-stimulating hormone; LH, luteinizing hormone; T, testosterone; PR, progressive; NP, non-progressive; IM, immotility; N, normal phenotype.

The two brothers with NOA and the NOA-affected sporadic cases were subjected to microsurgical testicular sperm extraction (micro-TESE) operation at the Center for Men’s Health, Urologic Medical Center, Shanghai General Hospital, and Shanghai Jiao Tong University School of Medicine. Histopathological analysis revealed SCOS phenotype in all three NOA-affected cases.

### WES Revealed Bi-Allelic *FANCM* Variants in the NOA-Affected Patients

**FIGURE 2:**
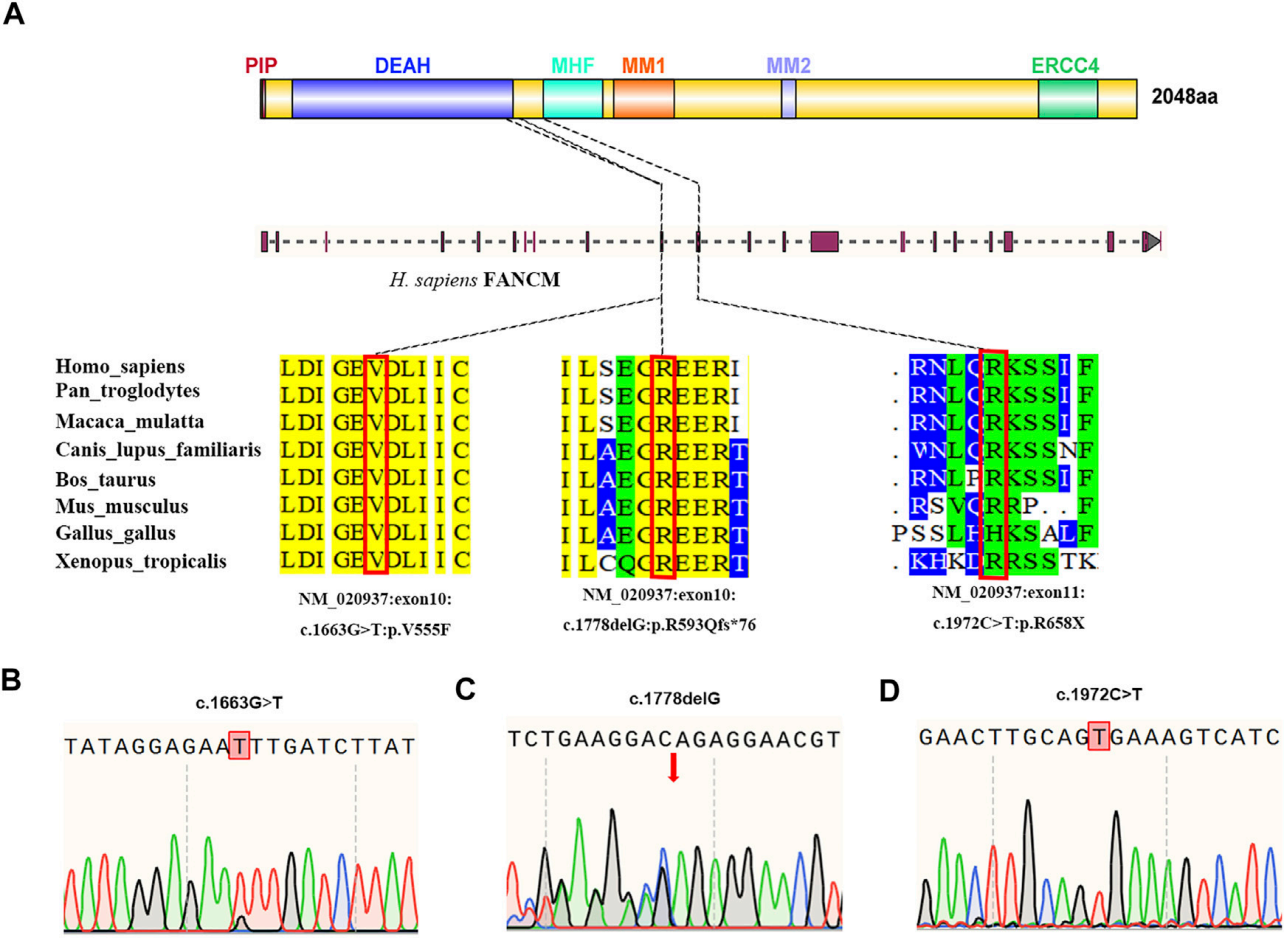
Structure of the FANCM protein and the genetic context of the *FANCM* variants detected in three cases diagnosed with NOA. **(A)** The positions of *FANCM* variants are shown and the conservation of the *FANCM* variants was analyzed. **(B–D)** Validation of *FANCM* variants identified by WES using Sanger sequencing in two brothers from the same family [**(B,C)**, P6649, and P6649-B] and a sporadic patient [**(D)**, P6612].

WES was performed in the two NOA-affected brothers (P6649 and P6649-B) to identify the potential PV for their male infertility. After the genetic analyses pipeline, compound heterozygous variants in *FANCM* (c. 1778delG: p. R593Qfs*76 and c. 1663G > T: p. V555F) were assumed as the most likely primary cause of spermatogenic defects in the two brothers (Figures 2A–C). Furthermore, we conducted WES in the 479 patients with NOA recruited from our center. Intriguingly, a sporadic individual (P6612) with a homozygous variant (c. 1972C > T: p. R658X) in *FANCM* was identified, and it was verified by Sanger sequencing (Figure 2D). Although this could be a homozygous LoF variant in *FANCM*, it is also possible that the individual carries a heterozygous R658X variant on the one allele and a heterozygous deletion in *FANCM* on the other allele. Thus, we employed the WES data for Copy Number Variant (CNV) analysis in this case according to the protocol as described previously (Yao et al., 2017; Marchuk et al., 2018; Tsuchida et al., 2018), and no evidence of *FANCM* deletion has been obtained in this NOA-affected case.

### The Impact of *FANCM* Variants

All three variants are extremely rare and are absent in the ExAC database and 1,000 Genomes Project database, respectively. It has been identified that FANCM contains six independent domains with separable functions so far, including PIP-box (aa 5–12), DEAH domain (aa 77–590), MHF binding domain (aa 661–800), MM1domain (aa 826–967), MM2 domain (aa 1,218–1,251), and ERCC4 domain (aa 1818–1956) (Domingues-Silva et al., 2019; O'Rourke et al., 2019). Correspondingly, the *FANCM* variants (c. 1778delG: p. R593Qfs*76 and c. 1972C > T: p. R658X) resulted in truncated FANCM proteins without expression of MHF binding domain, MM1 domain, MM2 domain, and ERCC4 domain. Furthermore, the variant (c. 1663G > T: p. V555F) was predicted to be deleterious by *in silico* analysis, including SIFT, PoyPhen-2, and MutationTaster analysis. Altogether, all three *FANCM* variants were assumed to be deleterious in the study, and the results are summarized in Table 2.

**TABLE 2:** Bi-allelic *FANCM* variants identified in the three NOA-effected cases.

Gene	Subject		
	P6649 and P6649-B		P6612
	*FANCM*	*FANCM*	*FANCM*
DNA change	c. 1663G > T	c. 1778delG	c. 1972C > T
Amino acid alteration	p. V555F	p. R593Qfs*76	p. R658X
Mutation type	compound heterozygous	compound heterozygous	homozygous
Allele Frequency in Human Populations			
East Asians in ExAC	0	0	0
1,000 Genomes Project	0	0	0
Functional Prediction			
SIFT	damaging	damaging	damaging
Polyphen-2	damaging	damaging	damaging
Mutation Taster	damaging	damaging	damaging

### SCOS Phenotype in the Subjects With Bi-Allelic Variants in *FANCM*

**FIGURE 3:**
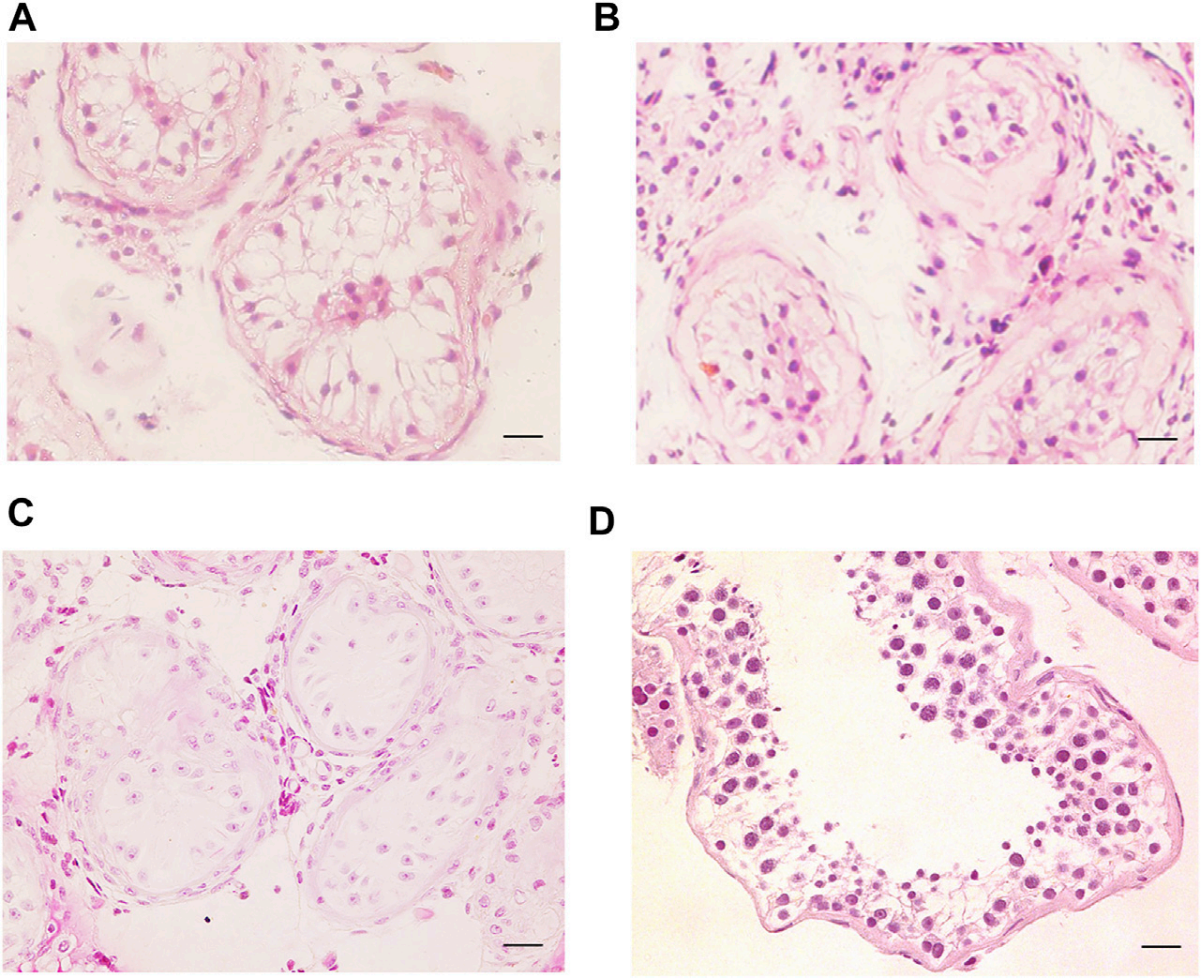
H&E staining of cross-sections of testis in NOA-affected patients. **(A–C)** H&E staining of cross-sections of testicular biopsy in the patient with NOA **(A)**, P6649; **(B)**, P6649-B; **(C)**, P6612). **(D)** H&E staining of cross-sections of seminiferous tubule in the OA-affected patient as the positive control. Scale bars = 20 μm in **(A–D)**.

**FIGURE 4:**
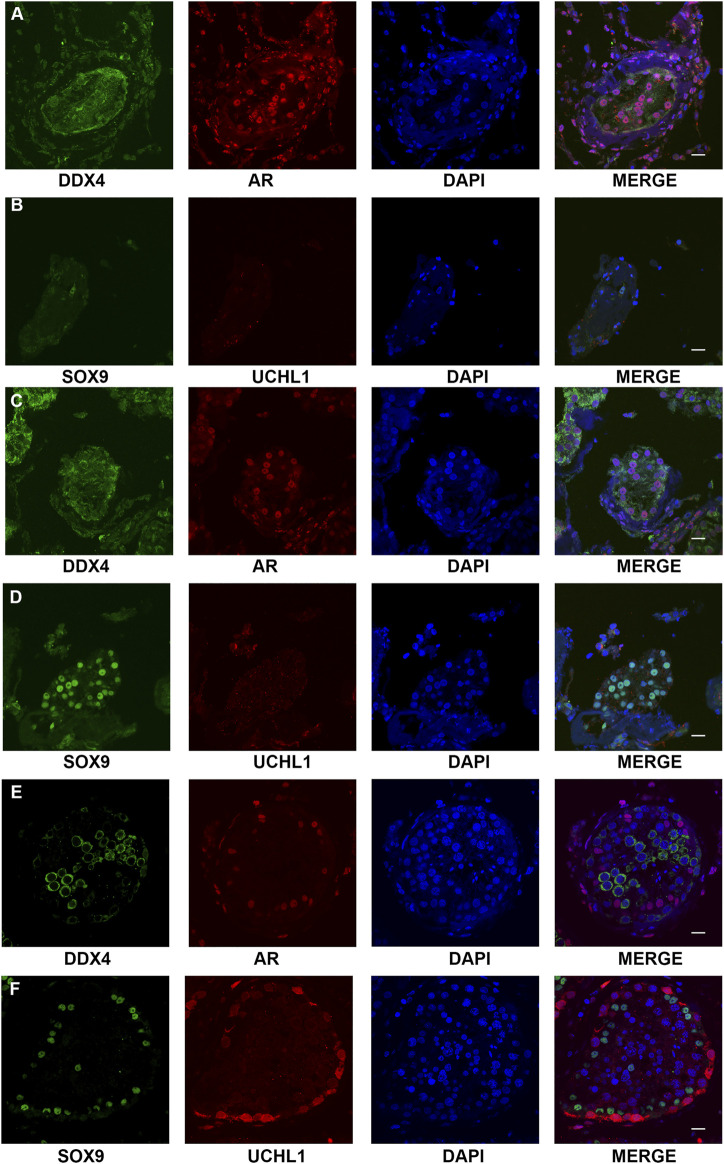
Expression of the markers of germ cells and somatic cells respectively in the testis of NOA-affected patients and patients with OA as the positive control. **(A–D)** IF staining results showed that there were no expressions of UCHL1 (a marker of spermatogonia) and DDX4 (hallmark of germ cells) in the testis of the proband **(A,B)** and his elder brother **(C,D)**, whereas all of the cells in seminiferous tubules were positive for AR or SOX9 (markers of Sertoli cells-affected). **(E,F)** IF staining results showed that there was normal spermatogenesis in the OA patients. Scale bars = 20 μm in **(A–E)**.

SCOS phenotype was verified in NOA-affected patients with bi-allelic *FANCM* variants using H&E and IF staining. H&E staining revealed that all germ cells were lost except for a single layer of Sertoli cells at the basement membrane within the seminiferous tubules of the two brothers (P6649 and P6649-B) with compound heterozygous *FANCM* variants (NM_020,937: c. 1778delG: p. R593Qfs*76) (Figures 3A,B). In contrast, there was normal spermatogenesis in the patients with OA as the positive control (Figure 3D). IF staining results showed that there were no expressions of UCHL1 (a marker of spermatogonia) and DDX4 (a hallmark of germ cells) in the testis of the two brothers, whereas all of the cells in seminiferous tubules were positive for AR or SOX9 (markers of Sertoli cells) (Figure 4). However, there was normal spermatogenesis in the OA-affected patients as shown by the positive signals of germ cells as well as somatic cells. Consistent with the two brothers in the pedigree, H&E staining also showed SCOS phenotype in the sporadic patient (P6612) with homozygous *FANCM* variant (NM_020937: c. 1972C > T: p. R658X) (Figure 3C). Altogether, these results suggested that bi-allelic variants in *FANCM* were associated with the SCOS phenotype.

## Discussion

In the present study, it is demonstrated that bi-allelic variants in *FANCM* could result in SCOS, and NOA. In the Chinese pedigree, compound heterozygous *FANCM* variants (c. 1778delG: p. R593Qfs*76 and c. 1663G > T: p. V555F) were identified in two NOA-affected brothers. Both variants were absent in the ExAC and 1,000 Genomes Project database. SIFT, PoyPhen-2, and MutationTaster analysis revealed that both variants were potentially deleterious. Furthermore, we identified another homozygous *FANCM* variant (c. 1972C > T: p. R658X) in a sporadic NOA-affected patient. SCOS phenotype was ascertained using H&E and IF staining in all three NOA-affected patients.

Fanconi Anemia (FA) is a rare genetic disease with bone marrow failure, organ defects, physical abnormalities, an increased risk of certain cancers, and cell hypersensitivity to DNA ICL-inducing agents (Deans and West, 2009; Neidhardt et al., 2017). FA core complex is composed of FANC-A, -B, -C, -E, -F, -G, -L, -M, FAAP24, and FAAP100 (Ciccia et al., 2007; Collis et al., 2008; Yang et al., 2013), which has been determined to play diverse and complex biological roles including involvement in DNA replication, repair, and in anti-crossover function to maintain genomic stability (Yan et al., 2010; Xue et al., 2015). Notably, FA mouse models show sub-fertile phenotypes in both genders, from reduced fertility to complete sterility (Tsui and Crismani, 2019), and suggesting that the FA pathway is associated with reproductive disorders.

FANCM is the most conserved member of the FA core complex, and it is reported that FANCM could affect different stages in male gametogenesis, including primordial germ cells (PGCs) proliferation, maintenance of spermatogonial stem cells (SSCs), and spermiogenesis. *Fancm*-deficient mice displayed a drastically decreased rate of proliferation of PGCs, a progressive reduction of SSCs, and partial maturation arrest at the round spermatid stage (Bakker et al., 2009; Yin et al., 2019). More seriously, some subsets of seminiferous tubules showed SCOS phenotype, and which is supposed to result from defective repair of ICL occurring in DNA replication of germ cells.

Consistent with the mice model, bi-allelic *FANCM* PV was also associated with both male and female infertility in humans. It was reported that females with bi-allelic LoF variants in *FANCM* presented with premature ovarian failure (POI) (Yang et al., 2020). Correspondingly, Kasak, *et al.* revealed that bi-allelic *FANCM* PV could result in SCOS (Kasak, et al., 2018). Recently, another study identified a homozygous *FANCM* PV in three brothers with idiopathic male infertility. Two suffered from oligoasthenospermia and another sporadic case was diagnosed with NOA (Yin et al., 2019). Similarly, in the current study, and novel variants in *FANCM* were identified in three patients with SCOS. However, none of our three patients has developed any systemic diseases or cancers like previous patients (Bogliolo et al., 2018; Yin et al., 2019). This discrepancy might be attributed to the difference of PV that is independent of the canonical FA pathway.

It is noteworthy that the age of the three NOA-affected patients in our study (P6649, 31 years; P6649-B, 34 years; P6612, 23 years) is younger than those in Bogliolo *et al.* Thus, future cancer incidence of the patients should be monitored.

FANCM protein has six critical functional domains including PIP-box, DEAH domain, MHF binding domain, MM1 domain, MM2 domain, and ERCC4 domain as mentioned above. Therein, the DEAH is the primary component that allows FANCM to transfer the core complex along with DNA during repair (Xue et al., 2015). It should be noted that the missense variant *FANCM* (c. 1663G > T) in this study is located in the DEAH domain, thus probably affecting the recruitment of the FA core complex. The MHF-FANCM complex could maintain genomic stability during cell division by inducing FACNM to bind to double-strand DNA and interact with chromatin (Yang et al., 2013). Furthermore, it has been reported that the MM1 domain could interact with FANCF within the FA core complex (Deans and West, 2009), and the MM2 domain could interact with RecQ-Mediated genome Instability protein 1 (RMI1) (Deans and West, 2009). In addition, the ERCC4 domain is necessary for the FAAP24 protein-binding function, and forming a heterodimer that is critical in protecting cells from ICL (Tao et al., 2012). Therefore, the LoF variants in *FANCM* (P6649 and P6649-B: c. 1778delG: p. R593Qfs*76; P6612: c. 1972C > T: p. R658X) may result in the instability of genome and a plethora of ICLs especially in PGCs and SSCs stages, which are supposed to be linked with the SCOS phenotypes of the three NOA-affected patients in our study. In short, the specific domain of FANCM accounts for different functions, and thus different PVs may cause the discrepancy of phenotypes. Moreover, the ICL repair pathway is indispensable in the DNA replication of germ cells, and can be affected by a variety of environmental agents, which have also been proposed as explanations for the clinical variability in male patients suffering from infertility.

However, there are also limitations in the current study. The missense variant *FANCM* (c. 1663G > T) in the two NOA-affected brothers, which is assumed to be deleterious by bioinformatics analysis, is located at the DEAH domain, and probably affecting the recruitment of the FA core complex. Thus, further studies should be performed to reveal the pathogenic mechanism of the variant (c. 1663G > T) through the *FANCM* ^*V555F*^ mutant mice model.

## Conclusion

In conclusion, we identified compound heterozygous variants in *FANCM* in two NOA-affected brothers and a homozygous LoF variant in *FANCM* in a sporadic case with NOA, respectively. H&E and immunohistochemistry showed SCOS in the three patients with NOA. Our study expands the knowledge of variants in *FANC*M and provides a new insight to understand the genetic etiology of NOA.

## Data Availability

The datasets for this article are not publicly available due to concerns regarding participant/patient anonymity. Requests to access the datasets should be directed to the corresponding authors
