# Supplementary material for: Novel Bi-Allelic Variants of FANCM Cause Sertoli Cell-Only Syndrome and Non-Obstructive Azoospermia
Source: Front Genet. 2021 Dec 15;12:799886. doi: 10.3389/fgene.2021.799886 (PMC8714797; doi:10.3389/fgene.2021.799886)
Supplement: Supplementary file 1 [file Table1.DOCX]

| **Table S1: The sequences of gene primers used for Sanger sequencing** | | | |
| --- | --- | --- | --- |
| Locus | Primer sequences (5’→3’) | | Product size (bp) |
| c.1663G>T | F | GTGTTGACATTACAGGCATGG | 448 |
| c.1663G>T | R | GGACTTGGAATGCTAACATG |  |
| c.1778delG | F | CAGTTTCGTGACGGTGGTTA | 522 |
| c.1778delG | R | AACTAAACACAAAGCACAGG |  |
| c.1972C>T | F | ATAATCAGAGTCAGTCCAAC | 529 |
| c.C1972C>T | R | AGCGCACATTGTAATCTGG |  |
