# Supplementary material for: Novel Bi-Allelic Variants of FANCM Cause Sertoli Cell-Only Syndrome and Non-Obstructive Azoospermia
Source: Front Genet. 2021 Dec 15;12:799886. doi: 10.3389/fgene.2021.799886 (PMC8714797; doi:10.3389/fgene.2021.799886)
Supplement: Supplementary file 2 [file DataSheet1.docx]

Novel Bi-allelic Variants of *FANCM* Cause Sertoli cell-only Syndrome and Non-obstructive Azoospermia

**Yuxiang Zhang^1†^, Peng Li^1^****^†^, Nachuan Liu^1†^, Tao Jing^1^, Zhiyong Ji^3^, Chao Yang^1^, Liangyu Zhao^1^, Ruhui Tian^1^, Huixing Chen^1^, Yuhua Huang^1^, Erlei Zhi^1^, Ningjing Ou^3^, Haowei Bai^1^, Yuchuan Zhou^2*^, Zheng Li^1^****^*^, Chencheng Yao^1*^**

^1^Department of Andrology, Center for Men’s Health, Department of ART, Institute of Urology, Urologic Medical Center, Shanghai Key Laboratory of Reproductive Medicine, Shanghai General Hospital, Shanghai Jiao Tong University School of Medicine, Shanghai 200080, China;

^2^The International Peace Maternity and Child Health Hospital, Shanghai Jiao Tong University School of Medicine, Shanghai, 200030, China;

^3^State Key Lab of Reproductive Medicine, Nanjing Medical University, Nanjing, 211100, China.

^†^These authors contributed equally to this work.

**^*^Correspondences:**

Chencheng Yao, Ph.D., Department of Andrology, Center for Men’s Health, Department of ART, Institute of Urology, Urologic Medical Center, Shanghai Key Laboratory of Reproductive Medicine, Shanghai General Hospital, Shanghai Jiao Tong University School of Medicine, Shanghai, China, Email: [yaochencheng@126.com](mailto:yaochencheng@126.com);

Zheng Li, M.D. & Professor, Department of Andrology, Center for Men’s Health, Department of ART, Institute of Urology, Urologic Medical Center, Shanghai Key Laboratory of Reproductive Medicine, Shanghai General Hospital, Shanghai Jiao Tong University School of Medicine, Shanghai, China, Email: [lizhengboshi@sjtu.edu.cn](mailto:lizhengboshi@sjtu.edu.cn);

Yuchuan Zhou, Ph.D. & Professor, The International Peace Maternity and Child Health Hospital, Shanghai Jiao Tong University School of Medicine, 145, Guang-yuan Road, Xuhui District, Shanghai, China, Email: [zhouych@sibcb.ac.cn](mailto:zhouych@sibcb.ac.cn).

The authors declare no conflict of interest.

The datasets used and/or analyzed during the current study available from the corresponding author, (Chencheng Yao), on reasonable request.
